# Latent profile analysis of oral health self-efficacy in maintenance hemodialysis patients and its impact on oral health

**DOI:** 10.3389/fpubh.2026.1867077

**Published:** 2026-07-15

**Authors:** Bixia Yuan, Yamei Guo, Weiling Yang, Jiran Tang, Miao Dong, Rongrong Wang, Yongmei Lu, Jieqian Wu, Yinqin Zhong

**Affiliations:** 1 Shenzhen Hospital (Futian) of Guangzhou University of Chinese Medicine, Shenzhen, Guangdong, China; 2 School of Nursing, Guangzhou University of Chinese Medicine, Guangzhou, Guangdong, China; 3 The Sixth Clinical Medical College of Guangzhou University of Traditional Chinese Medicine, Shenzhen, Guangdong, China; 4 Guangdong Provincial Hospital of Traditional Chinese Medicine University City Hospital, Guangzhou, Guangdong, China

**Keywords:** latent profile analysis, maintenance hemodialysis, oral health behaviors, oral health self-efficacy, oral health-related quality of life

## Abstract

**Objective:**

To investigate the heterogeneity of oral health self-efficacy among patients undergoing maintenance hemodialysis (MHD) and its influencing factors, and to compare the discrepancies in oral health-related quality of life (OHRQoL) across different patient subgroups, thereby furnishing evidence for the formulation of targeted interventions.

**Methods:**

A convenience sampling approach was adopted to recruit 352 MHD patients from three tertiary hospitals in Guangzhou from June 2023 to April 2024. Data were collected using a general information questionnaire, the Self-Efficacy Scale for Oral health (SESS), and the Oral Health Impact Profile-14 (OHIP-14). Latent profile analysis (LPA) was employed to identify latent classes of oral health self-efficacy. Univariate analysis and multivariate logistic regression analysis were performed to explore the influencing factors of class membership, and differences in OHRQoL among the classes were compared.

**Results:**

The total score of SESS was 49.60 ± 11.91, and that of the OHIP-14 was 12.61 ± 9.51. LPA identified three latent classes: C1 “low self-efficacy group” (17.3%), C2 “moderate self-efficacy group” (44.9%), and C3 “high self-efficacy group” (37.8%). The three-class model demonstrated optimal comprehensive fit indices and practical interpretability (Entropy = 0.966). Multivariate logistic regression analysis indicated that age, place of residence, monthly household income, number of comorbidities, daily tooth brushing frequency, and self-rated oral health status were significant influencing factors of oral health self-efficacy levels in MHD patients (all *p* < 0.05). There were statistically significant differences in OHRQoL scores among the different classes of MHD patients (*p* < 0.001). Improving oral health self-efficacy could effectively ameliorate patients’ OHRQoL.

**Conclusion:**

Oral health self-efficacy in MHD patients exhibits significant heterogeneity and can be categorized into three latent classes, with notable differences in OHRQoL across classes. Healthcare providers ought to develop class-specific stratified intervention strategies: focusing on improving basic oral care skills in the low self-efficacy group, correcting cognitive biases in the moderate self-efficacy group, and facilitating dental visit behaviors in the high self-efficacy group, so as to improve patients’ overall oral health-related quality of life.

## 1 Introduction

Maintenance hemodialysis (MHD) serves as an indispensable therapeutic modality for patients with end-stage renal disease (ESRD) to sustain renal function and maintain normal life. The global prevalence of hemodialysis patients is increasing at an annual rate of approximately 3%. With the widespread accessibility and optimized structure of medical insurance, the number of dialysis patients in China is increasing by roughly 10% per annum, and it is projected that by 2025, China will have approximately 1.34 million dialysis patients, accounting for approximately 27% of the global total (1). Improving the quality of life for patients with MHD has always been a key focus in medical health management. Among them, oral health has been listed by the World Health Organization as one of the top ten standards for overall health, closely related to overall health and quality of life. However, compared to the general population, the oral problems of MHD patients are more severe. Due to the restriction of fluid intake and the damage to salivary glands caused by uremia toxins, saliva secretion decreases and its composition changes, resulting in xerostomia and changes in taste in MHD patients (2). In the latest cross-sectional survey, the incidence of xerostomia was as high as 46.2, and 16.2% of the patients experienced changes in taste (3). Moreover, the oral mucosal lesions of MHD patients are particularly prominent, with 81.3% of the patients showing symptoms of pale oral mucosa, combined with the increase in urea levels in the patient’s body, the incidence of uremic halitosis can reach 78% (4). Not only that, oral frailty is also one of the specific frailty phenotypes of MHD patients, with an incidence rate of up to 45.2% (5). Affected by special immune deficiencies and metabolic disorders, the incidence of periodontal disease in MHD patients is higher, progresses faster, and is more difficult to control, reaching 36.3–99% (6). More importantly, the above oral problems interact with systemic pathological factors, affecting the prognosis of patients’ survival (7). The pathogens and toxins of chronic periodontitis can enter the patient’s bloodstream, activate systemic inflammatory responses, exacerbate protein energy consumption, and are independently associated with the patient’s all-cause mortality (8). A cohort study on MHD patients showed that the highest group of plaque index had a 3.04 times higher risk of all-cause mortality than the lowest two groups (9). The inflammatory state also affects cardiovascular health. Inflammatory factors can directly damage the vascular endothelium, accelerate the occurrence of cardiovascular events in MHD patients. At the same time, bacteria in the mouth are suspected to be significant in acute infections in MHD patients with low immune function (10). Finally, due to problems such as dry mouth and oral pain, patients’ difficulty in eating directly leads to insufficient protein and energy intake, exacerbating the vicious cycle of protein consumption and inflammation. This indicates that oral health is an indispensable part of the overall care for this group. However, the awareness of oral health care among patients with MHD is very poor, and there are deficiencies in their oral health behaviors (5).

Self-efficacy, a core construct of social cognitive theory proposed by Albert Bandura in the 1970s, refers to an individual’s confidence in their ability to perform a specific task or attain a particular goal. In the realm of oral health, oral health self-efficacy represents an important factor influencing oral hygiene behaviors and oral health status in MHD patients. A favorable level of oral health self-efficacy contributes to enhanced oral health behaviors and superior oral health outcomes (11, 12). Although numerous studies have explored oral health status and oral health awareness among MHD patients, there remains a paucity of research regarding the heterogeneity of oral health self-efficacy and targeted interventions in this patient cohort (13).

Oral health-related quality of life (OHRQoL) is a comprehensive indicator that assesses the extent to which oral diseases or conditions impair various aspects of an individual’s daily life, including functional limitations, pain and discomfort, psychological impact, and social interaction. Against the backdrop of the aforementioned severe oral health scenario, the overall OHRQoL of patients with ESRD has been compromised (14). Nevertheless, the pattern of OHRQoL may exhibit distinctive characteristics. For instance, one study found that despite a high objective burden of oral disease, OHRQoL scores in hemodialysis patients did not differ significantly from those of healthy individuals, whereas significant differences were observed when compared with kidney transplant recipients. Furthermore, no significant linear correlation was identified between various self-efficacy measures and OHRQoL among MHD patients (15). These findings underscore the complexity and heterogeneity within the MHD patient population, suggesting that traditional variable-centered analytical approaches may fail to fully elucidate the intrinsic relationship between oral self-efficacy and related quality of life in these patients. Additionally, given the prolonged disease course and high psychological burden of MHD patients, their self-efficacy exhibits displays marked heterogeneity, and conventional assessment and intervention methods may be inadequate in addressing the heterogeneous psychological needs of dialysis patients (16).

Latent profile analysis (LPA) is a statistical method that identifies subgroups of individuals with analogous characteristics based on their response patterns across continuous observed variables. In contrast to traditional methods, LPA can reveal within-population heterogeneity and facilitate the development of more precise interventions tailored to different subgroups (17). This method has been applied in the classification of health-related characteristics among patients with chronic diseases; however, in-depth exploration specifically focusing on oral health self-efficacy in MHD patients remains lacking (18, 19). Based on LPA, the present study aims to identify heterogeneous subgroups of oral health self-efficacy among MHD patients, clarify the characteristics and differences in oral health behaviors across these subgroups, and further compare their levels of oral health-related quality of life, thereby providing a basis for the formulation of stratified intervention strategies.

## 2 Methods

### 2.1 Study design

A cross-sectional study was conducted to assess Oral Health Self-Efficacy in Maintenance Hemodialysis Patients and its impact on Oral Health.

### 2.2 Study setting and participants

From June 2023 to April 2024, using the convenience sampling method, three tertiary grade A hospitals in Guangzhou, Guangdong Province, China were selected as the survey sites. These included one general hospital and two university-affiliated hospitals, aiming to enhance the representativeness of the institutions. A cross-sectional survey was conducted on the patients undergoing maintenance hemodialysis who met the criteria within each center. The inclusion criteria were defined as follows: (1) meeting the diagnostic criteria for end-stage renal disease (ESRD) according to the Guidelines for Screening, Diagnosis, and Prevention of Chronic Kidney Disease (20) and receiving MHD treatment with regular dialysis duration ≥ 3 months; (2) age ≥ 18; (3) clear consciousness, normal hearing and verbal expression abilities, and willingness and ability to cooperate with the survey; (4) informed consent to participate in this study. The exclusion criteria included: (1) concomitant with infectious diseases, malignant tumors, psychiatric disorders, or other severe organic diseases; (2) a history of kidney transplantation.

The sample size needs to be estimated based on the requirements of LPA and the study on influencing factors: each subgroup should have at least 50 samples (21). This study has 3 latent categories, so the minimum sample size should be 150 cases. For the study on influencing factors, a multivariate Logistic regression analysis is adopted. According to the rule of thumb, the sample size should be 5 to 10 times the number of independent variables (22). Considering a 20% dropout rate, in this study, we proposed 22 independent variables, which means the required sample size for this study should be at least 132 to 264 cases. Ultimately, a total of 352 participants were enrolled, which met the required sample size. This study was approved by the Ethics Committee of Guangdong Provincial Hospital of Traditional Chinese Medicine (Approval No. YE202311301).

### 2.3 Research tools

#### 2.3.1 General information questionnaire

A self-designed questionnaire was developed based on literature review and the study objectives. It included 15 items: sex, age, education level, place of residence, marital status, monthly household income, smoking status, primary disease, duration of hemodialysis, number of comorbidities, daily tooth brushing frequency, duration of each brushing, whether visited a dentist within the past year, self-rated oral health status, and perceived dental treatment needs.

#### 2.3.2 Self-efficacy scale for self-care (SESS)

This scale was originally developed by Kakudate et al. (23) and translated into Chinese by Wu et al. (24). The scale consists of three dimensions (dental visit self-efficacy, correct tooth brushing self-efficacy, and balanced diet self-efficacy) with 15 items. Each item is scored from 1 (“not confident at all”) to 5 (“very confident”), with total scores ranging from 15 to 75. Higher scores indicate stronger oral health self-efficacy. In this study, the Cronbach’s *α* coefficient of the scale was 0.835.

#### 2.3.3 Oral health impact profile-14 (OHIP-14)

The Chinese version of OHIP-14, revised by Xin and Lin (25), was used. The scale includes four dimensions with 14 items. Each item is scored from 0 (“never”) to 4 (“always”), with higher scores indicating poorer oral health-related quality of life (OHRQoL). In this study, a cutoff of 12 on the OHIP-14 was used: scores ≥ 12 indicated poor OHRQoL, and scores <12 indicated good OHRQoL (26). The Cronbach’s α coefficient of the scale in this study was 0.904.

### 2.4 Data collection

Data were analyzed using SPSS 25.0 and Mplus 8.3. Model fit indices for LPA included Akaike Information Criterion (AIC), Bayesian Information Criterion (BIC), sample-adjusted BIC (aBIC), entropy, Lo–Mendell–Rubin adjusted likelihood ratio test (LMRT), and bootstrap likelihood ratio test (BLRT). Lower AIC, BIC, and aBIC values, higher entropy (entropy >0.80 indicates classification accuracy>90%), and significant LMRT and BLRT indicate better model fit (27). Categorical data were presented as frequencies and percentages, and continuous data as means ± standard deviations. Comparisons among multiple groups were performed using chi-square tests or adjusted chi-square tests. Influencing factors were analyzed using multivariate logistic regression. The significance level was set at *α* = 0.05.

### 2.5 Statistical methods

Data were double-checked, organized, and entered into a database. Latent profile modeling was conducted in M plus 8.3 using the 5 TCM-HL dimensions as observed variables. Models with increasing class numbers were evaluated, and the optimal solution was selected based on both model fit and clinical comprehensibility. The evaluation criteria included: (1) Information criteria—AIC, BIC, and aBIC, where lower values indicate a better fit; (2) Classification accuracy—Entropy values (ranging from 0 to 1), with values closer to 1 indicating higher accuracy; and (3) Likelihood ratio tests-Lo-Mendell-Rubin (LMR) and bootstrap likelihood ratio test (BLRT), where a *p*-value<0.05 suggests that the K-class model outperforms the K − 1-class model. Common method bias was assessed using Harman’s single-factor test in SPSS 27.0. Skewness and kurtosis tested univariate normality; Mardia’s test assessed multivariate normality. Variables with a normal or approximately normal distribution are reported as Mean ± SD and compared using one-way ANOVA. Categorical variables are presented as frequencies or percentages and analyzed using the chi-square test or Fisher’s exact test. Variables that are significant in univariate analysis are included in multivariate logistic regression. A *p*-value of <0.05 is considered statistically significant.

## 3 Results

### 3.1 General information of participants

A total of 361 questionnaires were distributed, and 352 valid questionnaires were returned, yielding an effective response rate of 97.5%. The general information of the participants is shown in Table 1.

**Table 1:** General information of maintenance hemodialysis patients (*n* = 352).

Variables		Number	(%)
Gender	Male	209	59.4
	Female	143	40.6
Age	18 ~ 45	59	16.8
	46 ~ 60	136	38.6
	>60	157	44.6
Education level	Primary school and below	110	31.3
	Junior high school	134	38.1
	High school or technical secondary school	60	17.0
	Junior college	22	6.3
	Bachelor degree and above	26	7.4
Residence	Rural	80	22.7
	County	129	36.6
	City	143	40.6
Marital status	Unmarried	20	5.7
	Married	276	78.4
	Divorced/Widowed	56	15.9
Family per-capita monthly income (RMB)	<1,000	19	5.4
	1,000 ~ 3,000	85	24.1
	3,000 ~ 5,000	109	31.0
	>5,000	139	39.5
Smoking	Yes	97	27.6
	No	255	72.4
Original diseases	Chronic glomerulonephritis	127	36.1
	Diabetes	107	30.4
	Hypertension	87	24.7
	Others	31	8.8
Years of hemodialysis	<1	94	26.7
	1 ~ 3	140	39.8
	3 ~ 10	100	28.4
	>10	18	5.1
Number of combined diseases	<3	280	79.5
	≥3	72	20.5
Times of brushing teeth	Never or occasionally	35	9.9
	Once a day	155	44.1
	≥Twice a day	162	46.0
The duration of brushing teeth each time	<3 min	213	60.5
	≥3 min	139	39.5
Have seen a dentist in the past year	Yes	88	75.0
	No	264	25.0
Self-rated oral health status	Worse	122	34.7
	General	152	43.1
	Well	78	22.2
Perceived dental treatment need	Yes	182	51.7
	No	170	48.3

### 3.2 Latent profile analysis and profile naming of oral health self-efficacy in maintenance hemodialysis patients

**Figure 1:**
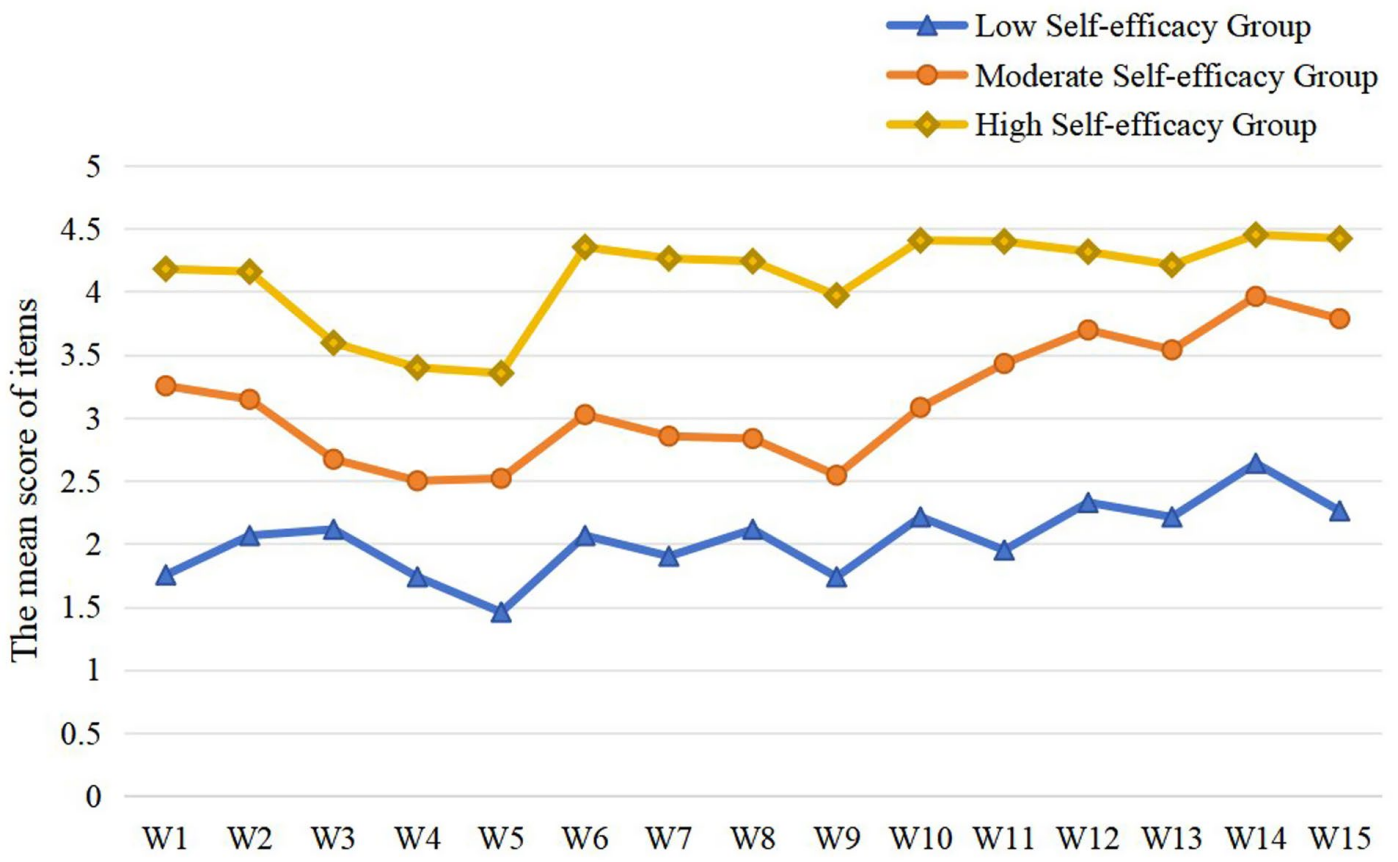
Distribution of three potential categories of self-efficacy for oral care in MHD patients.

Latent profile models were constructed using the 15 items of the oral health self-efficacy scale as manifest variables. The model fit indices are summarized in Table 2. AIC, BIC, and aBIC gradually decreased as the number of classes increased, indicating better model fit with more classes. When three classes were retained, the entropy value was 0.966, and both the LMRT and BLRT were statistically significant (*p* < 0.05). Moreover, the average posterior probability of belonging to each class exceeded 90% for all three classes. When four or five classes were retained, the entropy value decreased and the LMRT increased. Based on a combination of model fit indices and practical interpretability, the three-class model was selected. A latent profile plot was drawn based on the classification results (see Figure 1). The three classes were named according to the mean scores of the items. Class C1 accounted for 17.3% of the sample, Class C2 for 44.9%, and Class C3 for 37.8%. The mean item scores of the three classes were low, moderate, and high, respectively. Specifically, Class C1 had generally low mean scores on all items and was named the low self-efficacy group, consisting of 61 cases (17.3%). Class C2 had mean scores between those of Class C1 and Class C3 and was named the moderate self-efficacy group, consisting of 158 cases (44.9%). Class C3 had generally high mean scores and was named the high self-efficacy group, consisting of 133 cases (37.8%).

**Table 2:** Fitting results of the potential profile model of self-efficacy for oral care level in MHD patients.

Model	AIC	BIC	aBIC	Entropy	P		Class probability
					LMRT	BLRT	
1	15784.862	15900.771	15805.599	—	—	—	1
2	13774.787	13952.514	13806.584	0.910	0.073	<0.001	0.474/0.526
3	12918.367	13157.912	12961.224	0.966	<0.001	<0.001	0.173/0.449/0.378
4	12673.505	12974.868	12727.422	0.953	0.086	<0.001	0.168/0.369/0.298/0.165
5	12435.399	12798.631	12500.426	0.947	0.049	<0.001	0.170/0.122/0.310/0.253/0.145

### 3.3 Oral health self-efficacy scores across different classes of maintenance hemodialysis patients

The total score of oral health self-efficacy in maintenance hemodialysis (MHD) patients was 49.60 ± 11.91. Significant differences in oral health self-efficacy scores were observed across the three classes. The low self-efficacy group (Class C1) had the lowest scores in dental visit self-efficacy (8.92 ± 2.69), tooth brushing self-efficacy (10.03 ± 2.69), balanced diet self-efficacy (11.39 ± 2.26), and total score (30.34 ± 4.13). In contrast, the moderate self-efficacy group (Class C2) showed higher scores in dental visit self-efficacy (14.09 ± 3.45), tooth brushing self-efficacy (14.34 ± 2.60), balanced diet self-efficacy (18.41 ± 2.13), and total score (46.84 ± 4.21). The high self-efficacy group (Class C3) had the highest scores in dental visit self-efficacy (18.68 ± 2.50), tooth brushing self-efficacy (21.23 ± 2.45), balanced diet self-efficacy (21.80 ± 2.38), and total score (61.71 ± 4.49) (see Table 3).

**Table 3:** Oral health care efficacy scores among maintenance hemodialysis patients of different categories (*n* = 352).

Group	*N*	Self-efficacy for dentist consultations	Self-efficacy for brushing of the teeth	Self-efficacy for dietary habits	Total scores
C1	61	8.92 ± 2.69	10.03 ± 2.69	11.39 ± 2.26	30.34 ± 4.13
C2	158	14.09 ± 3.45	14.34 ± 2.60	18.41 ± 2.13	46.84 ± 4.21
C3	133	18.68 ± 2.50	21.23 ± 2.45	21.80 ± 2.38	61.71 ± 4.49

C1, low self-efficacy group; C2, moderate self-efficacy group; C3, high self-efficacy group.

### 3.4 Univariate analysis of latent classes of oral health self-efficacy in maintenance hemodialysis patients

Univariate analysis was conducted with the three latent classes as the dependent variable and general characteristics as independent variables. The results showed that age, education level, place of residence, monthly household income per capita, primary disease, number of comorbidities, daily tooth brushing frequency, duration of each brushing, and self-rated oral health status were statistically significant factors associated with the latent classes of oral health self-efficacy (all *p* < 0.05) (see Table 4 for details).

**Table 4:** Single factor analysis of three potential categories of self-efficacy for oral care in MHD patients (*n*, %).

Variables	Number (*n* = 352)	C1 (*n* = 61)	C2 (*n* = 158)	C3 (*n* = 133)	*χ* ^2^	*p*
Gender					0.442^a^	0.822
Male	209(59.4)	37(60.7)	96 (60.8)	76 (57.1)		
Female	143 (40.6)	24 (39.3)	62 (39.2)	57 (42.9)		
Age (years)					12.436^a^	0.014
18 ~ 45	59 (16.8)	13 (21.3)	20 (12.7)	26 (19.5)		
46 ~ 60	136 (38.6)	19 (31.1)	55 (34.8)	62 (46.6)		
>60	157 (44.6)	29 (47.5)	83 (52.5)	45 (33.8)		
Education level					28.018^a^	<0.001
Primary school and below	110 (31.3)	28 (45.9)	54 (34.2)	28 (21.1)		
Junior high school	134 (38.1)	22 (36.1)	67 (42.4)	45 (33.8)		
High school or technical secondary school	60 (17.0)	6 (9.8)	23 (14.6)	31 (23.3)		
Junior college	22 (6.3)	3 (4.9)	8 (5.1)	11 (8.3)		
Bachelor degree and above	26 (7.4)	2 (3.3)	6 (3.8)	18 (13.5)		
Residence					17.432^a^	0.002
Rural	80 (22.7)	19 (31.1)	44 (27.8)	17 (12.8)		
County	129 (36.6)	22 (36.1)	61 (38.6)	46 (34.6)		
City	143 (40.6)	20 (32.8)	53 (33.5)	70 (52.6)		
Marital status					4.175^b^	0.400
Unmarried	20 (5.7)	4 (6.6)	5 (3.2)	11 (8.3)		
Married	276 (78.4)	48 (78.7)	125 (79.1)	103 (77.4)		
Divorced/Widowed	56 (15.9)	9 (14.8)	28 (17.7)	19 (14.3)		
Family per-capita monthly income (RMB)					39.687^b^	<0.001
<1,000	19 (5.4)	8 (13.1)	7 (4.4)	4 (3.0)		
1,000 ~ 3,000	85 (24.1)	21 (34.4)	48 (30.4)	17 (12.0)		
3,000 ~ 5,000	109 (31.0)	18 (29.5)	54 (34.2)	38 (27.8)		
>5,000	139 (39.5)	14 (23.0)	49 (31.0)	78 (57.1)		
Smoking					0.348^a^	0.843
Yes	97 (27.6)	16 (26.2)	46 (29.1)	35 (26.3)		
No	255 (72.4)	45 (73.8)	112 (70.9)	98 (73.7)		
Original diseases					19.758^a^	0.003
Chronic glomerulonephritis	127 (36.1)	14 (23.0)	55 (34.8)	58 (43.6)		
Diabetes	107 (30.4)	30 (49.2)	50 (31.6)	27 (20.3)		
Hypertension	87 (24.7)	13 (21.3)	42 (26.6)	32 (24.1)		
Others	31 (8.8)	4 (6.6)	11 (7.0)	16 (12.0)		
Years of hemodialysis					4.240^b^	0.657
<1	94 (26.7)	13 (21.3)	43 (27.2)	38 (28.6)		
1 ~ 3	140 (39.8)	27 (44.3)	68 (43.0)	45 (33.8)		
3 ~ 10	100 (28.4)	18 (29.5)	40 (25.3)	42 (31.6)		
>10	18 (5.1)	3 (4.9)	7 (4.4)	8 (6.0)		
Number of combined diseases					53.313^a^	<0.001
<3	280 (79.5)	28 (45.9)	132 (83.5)	120 (90.2)		
≥3	72 (20.5)	33 (54.1)	26 (16.5)	13 (9.8)		
Times of brushing teeth					68.611^a^	<0.001
Never or occasionally	35 (9.9)	14 (23.0)	18 (11.4)	3 (2.3)		
Once a day	155 (44.1)	31 (50.8)	87 (55.1)	37 (27.8)		
≥Twice a day	162 (46.0)	16 (26.2)	53 (33.5)	93 (69.9)		
The duration of brushing teeth each time					26.951^a^	<0.001
<3 min	213 (60.5)	47 (77.0)	108 (68.4)	58 (43.6)		
≥3 min	139 (39.5)	14 (23.0)	50 (31.6)	75 (56.4)		
Have seen a dentist in the past year					3.692^a^	0.155
Yes	88 (25.0)	19 (31.1)	32 (20.3)	37 (27.8)		
No	264 (75.0)	42 (68.9)	126 (79.7)	96 (72.2)		
Self-rated oral health status					83.496^a^	<0.001
Worse	122 (34.7)	46 (73.8)	56 (35.4)	21 (15.8)		
General	152 (43.1)	15 (23.0)	80 (50.6)	58 (43.6)		
Well	78 (22.2)	2 (3.2)	22 (13.9)	54 (40.6)		
Perceived dental treatment need					3.877^a^	0.144
Yes	182 (51.7)	38 (62.3)	75 (47.5)	69 (51.9)		
No	170 (48.3)	23 (37.7)	83 (52.5)	64 (48.1)		

C1, low self-efficacy group; C2, moderate self-efficacy group; C3, high self-efficacy group.

a Chi-square test.

b Adjusted chi-square test.

### 3.5 Multinomial logistic regression analysis of latent classes of oral health self-efficacy in maintenance hemodialysis patients

A multinomial logistic regression analysis was performed with the three latent classes of oral health self-efficacy as the dependent variable and the statistically significant factors from univariate analysis as independent variables. The results showed that age (18–45 years = 1; 46–60 years = 2; >60 years = 3, with >60 years as reference), place of residence (rural = 1; county = 2; urban = 3, with urban as reference), monthly household income per capita (<1,000 CNY = 1; 1,000–3,000 CNY = 2; 3,000–5,000 CNY = 3; >5,000 CNY = 4, with >5,000 CNY as reference), number of comorbidities (<3 = 1; ≥3 = 2, with ≥3 as reference), daily tooth brushing frequency (never or occasionally = 1; once a day = 2; ≥2 times a day = 3, with ≥2 times a day as reference), and self-rated oral health status (poor = 1; fair = 2; good = 3, with good as reference) significantly influenced the latent class membership of oral health self-efficacy in MHD patients (see Table 5).

**Table 5:** Multinomial logistic regression analysis of potential categories of self-efficacy for oral care in MHD patients (*n* = 352).

Variables	*β*	SE	Wald χ^2^	*p*-value	OR	95%CI
C1 vs. C2						
Intercept	2.009	1.384	2.107	0.147		
Number of combined diseases						
<3	1.559	0.390	16.004	<0.001	4.752	2.214 ~ 10.197
Self-rated oral health status						
Worse	−1.721	0.824	4.366	0.037	0.179	0.036 ~ 0.899
C1 vs. C3						
Intercept	−3.584	1.432	6.263	0.012		
Family per-capita monthly income (RMB)						
1,000 ~ 3,000	1.416	0.592	5.716	0.017	4.119	1.291 ~ 13.147
Number of combined diseases						
<3	−2.137	0.496	18.601	<0.001	0.118	0.045 ~ 0.312
Times of brushing teeth						
Never or occasionally	2.571	0.851	9.118	0.003	13.077	2.465–69.382
Once a day	1.258	0.457	7.578	0.006	3.517	1.437 ~ 8.612
Self-rated oral health status						
Worse	2.965	0.851	12.147	<0.001	19.397	3.661 ~ 102.774
C2 vs. C3						
Intercept	−1.576	0.837	3.543	0.060		
Age						
46 ~ 60	−0.666	0.323	4.254	0.039	0.514	0.273 ~ 0.967
Residence						
Rural	1.053	0.427	6.089	0.014	2.866	1.242 ~ 6.613
Family per-capita monthly income(RMB)						
1,000 ~ 3,000	1.214	0.424	8.176	0.004	3.366	1.465 ~ 7.734
Times of brushing teeth						
Never or occasionally	1.919	0.728	6.950	0.008	6.814	1.636 ~ 28.376
Once a day	1.105	0.301	13.499	<0.001	3.020	1.675 ~ 5.447
Self-rated oral health status						
Worse	1.244	0.427	8.472	0.004	3.470	1.501 ~ 8.021
General	0.860	0.362	5.664	0.017	2.364	1.164 ~ 4.802

Likelihood ratio *χ*^2^ = 186.114, *p* < 0.001; goodness-of-fit test *p* > 0.05, maximum.

### 3.6 Comparison of total and domain scores of oral health-related quality of life across different oral health self-efficacy classes in maintenance hemodialysis patients

Using the three classes of oral health self-efficacy as grouping variables, univariate analysis was performed on oral health-related quality of life (OHRQoL). The results showed that there were statistically significant differences in all domain scores (physical functional limitation, pain and discomfort, psychological impact, and reduced functional independence) and the total score of OHRQoL among the three groups of MHD patients (*p* < 0.001). Further pairwise comparisons using the least significant difference (LSD-t) method indicated that the domain scores and total score of OHRQoL followed the order: Class C1 > Class C2 > Class C3 (all *p* < 0.001) (see Table 6 for details).

**Table 6:** Comparison of oral health-related quality of life in MHD patients with different self-efficacy for oral care categories (*n* = 352).

Group	Pain and discomfort	Psychological discomfort	Functional limitation	Handicap	Total scores
C1	6.70 ± 2.20	4.90 ± 2.23	6.28 ± 2.08	8.15 ± 3.53	26.03 ± 8.27
C2	4.19 ± 1.97	2.30 ± 1.75	3.43 ± 1.89	3.53 ± 2.54	13.46 ± 6.25
C3	2.08 ± 2.00	1.02 ± 1.44	1.26 ± 1.53	1.09 ± 1.73	5.45 ± 5.23
*F*	113.962	104.770	167.959	168.808	225.741
*p*	<0.001	<0.001	<0.001	<0.001	<0.001
Pairwise comparisons (*p* < 0.001)	C1 > C2 > C3	C1 > C2 > C3	C1 > C2 > C3	C1 > C2 > C3	C1 > C2 > C3

C1, low self-efficacy group; C2, moderate self-efficacy group; C3, high self-efficacy group.

## 4 Discussion

### 4.1 Oral health self-efficacy levels can be classified into three latent profiles

This study found that oral health self-efficacy levels among maintenance hemodialysis (MHD) patients can be classified into three latent class structures: C1: low self-efficacy group; C2: moderate self-efficacy group; and C3: high self-efficacy group.

Class C1 accounted for 17.3% of the total sample and exhibited generally low self-efficacy levels (30.34 ± 4.13). These patients obtained the lowest scores across the dimensions of oral health knowledge, skills, and confidence, and their performance in oral health behaviors was significantly lower than that of the other classes. Patients in this group scored the lowest in terms of knowledge, skills, and confidence regarding oral health care. Moreover, the implementation rate of oral health behaviors was significantly lower than that of other groups. In terms of oral health behaviors, their implementation rate was significantly lower. Half of the patients brushed their teeth only once a day, and 77% of the patients spent less than 3 min each time brushing. Regarding dental service needs, 62.3% of the patients in Class C1 indicated a need for dental treatment, but only 31.1% had visited a dentist within a year, presenting a contradiction of “high demand but low utilization.” The majority of these patients were from rural areas, with low educational levels, and had low household income. On the one hand, economic factors such as the proportion of medical expenses significant determinants of oral health status in this population (28). On the other hand, individual health literacy plays an important role: MHD patients with higher educational levels actively seek oral health-related knowledge and maintain good oral hygiene habits (29). Conversely, patients with a lower level of education have more limited access to oral health care knowledge and channels, and are more difficult to cope with oral problems. The disparity in health resources and educational background jointly restricts the ability of this group of people to obtain, understand, and apply oral health information, leading to low self-efficacy and further solidifying poor oral health behaviors and medical-seeking habits.

Class C2 accounted for 44.9% of the total sample, with self-efficacy scores falling between those of Class C1 and Class C3 (46.84 ± 4.21). Their performance in oral health behaviors was better than that of the Class C1, but there were also significant shortcomings. 55.1% of the patients brushed their teeth only once a day, and 68.4% of the patients spent less than 3 min each time brushing. Most participants were capable of maintaining a certain degree of autonomy in dietary management, yet their performance in oral health behaviors remained markedly inadequate. A high proportion of patients in this class rated their oral health as “fair” or “poor.” Nevertheless, they scored-relatively high on the balanced diet dimension, indicating that while they paid attention to general health and diet, their emphasis on and practical engagement with oral health were still insufficient. In terms of dental treatment needs, 47.5% of the patients indicated a need for it. However, the proportion of those who had visited a dentist within the past year was only 20.3%, presenting the characteristic of “moderate demand but low utilization.” Their health awareness was confined to the framework of systemic health and had not established a direct psychological connection with oral health. They might focus on dietary health due to their physical illness, yet overlook the concomitant oral problems, resulting in a failure to translate their awareness from dietary management to oral hygiene behaviors. From a cognitive psychology perspective, this phenomenon may be associated with compensatory health beliefs (CHBs), defined as the cognitive tendency to believe that the negative effects of unhealthy behaviors can be offset by other health-promoting behaviors (30). Among the Class C2 patients in this study, there might be an implicit compensatory belief: they may hold the view that good performing in dietary management can “compensate” for or “offset” the negative consequences of inadequate oral health maintenance. Consequently, they concentrate their health management efforts on dietary control, which is directly related to dialysis outcomes, while treating oral health as a secondary, compensable domain. Moreover, CHBs often operate through a moral licensing mechanism, whereby “good performance” in one health domain provides psychological permission to slack off in another domain. This precisely explains the knowledge–behavior gap observed in Class C2 patients (31).

Class C3 accounted for 37.8% of the total sample, with high self-efficacy across all dimensions (61.71 ± 4.49). Among them, 69.9% brushed their teeth at least twice daily, and 40.6% rated their oral health as good. However, the proportion of patients in this class who had visited a dentist within the past year was not prominent, presenting a cognitive-behavioral disconnect characterized by “high self-efficacy but low dental utilization.” This finding is consistent with the study by Xie et al. (32), which reported that only 21.7% of patients had visited a dental clinic in the past year, with many using “no toothache” as the criterion for oral health; they might experience oral problems but do not seek regular care, neglecting preventive treatment. These individuals may be overconfident in their self-management abilities, or they may be constrained by objective factors such as cost, time, and the accessibility of dental services, which impede dental visits. From the perspective of the Health Belief Model, first, the strength of the association between self-efficacy and different oral health behaviors varies. Studies have shown that self-efficacy is the strongest predictor of tooth brushing behavior (33, 34), but its association with dental visiting behavior is not significant. This explains why Class C3 patients, despite having good brushing habits, still had a low rate of dental visits. Second, perceived barriers are a key predictor of dental visiting behavior and exert a strong negative predictive effect: the higher the perceived barriers, the lower the likelihood of a dental visit (34). Although Class C3 patients had high self-efficacy, they might still be limited by objective perceived barriers such as time costs, accessibility, and financial burden, thereby failing to initiate dental visiting behavior.

### 4.2 Factors influencing latent class membership of oral health self-efficacy in MHD patients

This study found that age, place of residence, income, number of comorbidities, tooth brushing habits, and self-rated oral health status significantly influenced latent class membership of oral health self-efficacy in MHD patients.

#### 4.2.1 Individual characteristics

Patients in different classes exhibited significant differences. Socioeconomic factors and health resources constitute important material support dimensions for oral health self-efficacy. The majority of Class C2 patients resided in rural areas, suggesting that rural regions may face practical challenges such as poor accessibility of oral health resources and insufficient coverage of oral health education (35). Moreover, the monthly family income of patients in the Class C1 and Class C2 was relatively low, indicating that economic pressure might directly limit the patients’ ability to access oral hygiene products and professional services (36). Existing studies have shown that low-income families have significantly restricted access to oral hygiene products such as toothbrushes and fluoride toothpaste, as well as basic oral health services (37), further affecting their confidence and behavior in investing in oral health. From the perspective of age composition, the patients in the Class C1 and Class C2 were mainly older adult individuals (accounting for 47.5 and 52.5% respectively), and older adult individuals might face problems such as decreased physical function, fixed health concepts, and weakened ability to obtain oral health information (38), which further explains the low self-efficacy of these two groups. Secondly, age group and health awareness might also play a role through social support mechanisms. Groups dominated by older adult individuals often face higher risks of family instability, and a study has pointed out that nearly half of the older adults MHD patients’ families have a persistently low level of resilience (39). Additionally, the family structure of rural older adult individuals in China is more vulnerable and prone to changes due to individual characteristics and the status of their children (40). The patients in the Class C3 were typically aged between 46 and 60 years old, different from the older adult individuals with a higher proportion in the Class C1 and Class C2. Middle-aged patients might be able to maintain good oral hygiene habits due to stronger health awareness and more stable living conditions. One study tracked changes in oral health-related quality of life from adulthood to middle age and found that oral self-care habits remained stable during middle age (41). In addition, research has shown that social support can effectively enhance individual self-efficacy, and the improvement of self-efficacy is positively correlated with the improvement of health behaviors (42). In the present study, the relatively stable family relationships of middle-aged patients in Class C3 provided them with continuous emotional support and behavioral reminders, while a relatively solid economic foundation reduced barriers to accessing oral health products and professional services. These external support resources collectively contributed to enhancing their self-efficacy in oral health. This finding further suggests that clinical practitioners should pay attention to patients’ social support systems and promote the long-term maintenance of health behaviors by enhancing self-efficacy.

Our study also found that the number of comorbidities influenced oral health self-efficacy. Contrary to expectations, patients with fewer comorbidities did not uniformly exhibit high self-efficacy but instead exhibited a “bipolar” pattern, with both Class C3 and Class C1 containing such patients. This finding is consistent with the results observed by Lee and Lee (43) in patients with comorbid hypertension. Previous studies have indicated that comorbidities such as diabetes or hypertension significantly deteriorate objective oral health indicators in hemodialysis patients, including periodontal status and saliva secretion (44). From the perspective of the Health Belief Model, this phenomenon can be attributed to differences in individual illness perception. When the objective disease burden is relatively low, some patients may not yet perceive a direct threat to their oral health from comorbidities, leading to low perceived susceptibility and perceived severity. They may underestimate oral health risks and lower the priority of self-care, believing that “teeth are fine,” thereby exhibiting low self-efficacy. In contrast, other patients with fewer comorbidities may possess sufficient self-management resources and confidence because of their overall better health status, thus maintaining high-efficacy health behaviors and showing high self-efficacy. This aligns with the findings of Paulus et al. (45), who reported that differences in illness perception lead to significant differentiation in self-efficacy. This suggests that healthcare providers should dynamically assess patients’ health beliefs and illness perception levels to identify potential behavioral risks and implement targeted interventions.

#### 4.2.2 Cognitive-behavioral level

The study found that self-rated oral health status had a complex “double-edged sword” effect on self-efficacy class membership: it could either motivate some patients to adopt more proactive dietary management or be associated with extremely low self-efficacy and behavioral adherence. Patients in Class C2 who rated their oral health as poor had a significantly increased risk of belonging to Class C1, indicating that subjective negative evaluation of oral health may lead to distinctly different behavioral responses. On the one hand, poor self-rating might arouse health awareness in some patients, prompting them to take action in relatively easier domains such as dietary management. Mohammadkhah et al. (46) showed that increasing perceived threat can effectively trigger health behavior change. The Class C2 patients in this study may have formed a “threat perception” from negative evaluations of their oral condition, thereby initiating health management behaviors. On the other hand, poor self-rating might also lead to psychological defensive avoidance. Jia et al. found that when health information triggers negative emotions such as anxiety or frustration, individuals tend to adopt information avoidance strategies to escape the information maze (47). When faced with the negative self-evaluation of “poor oral condition,” the patients in this study might experience excessive risk perception or cognitive overload. For example, in managing multiple chronic diseases, patients may become cognitively overloaded and unable to cope with the growing demand for health information, thereby selectively abandoning oral health behaviors, leading to decreased self-efficacy. It is worth noting that the effect of perceived threat does not operate in isolation. The study by Mohammadkhah et al. (46) also indicated that behavior change is moderated by perceived barriers and self-efficacy. The reason why Class C2 patients remained at the level of “dietary management” without extending to comprehensive oral hygiene behaviors may be that they perceived higher barriers to oral health (e.g., time-consuming brushing, difficulty using dental floss), resulting in incomplete translation of behavioral intentions into actions. Furthermore, daily tooth brushing frequency was also a risk factor for low oral health self-efficacy, consistent with the findings of Bantel et al., which indicated that oral health self-efficacy is significantly associated with high-frequency brushing (≥3 times/day) (48).

In this study, the self-efficacy of MHD patients was categorized into three types: C1 (low efficacy), C2 (moderate efficacy), and C3 (high efficacy). There were significant differences in the oral health behavior patterns corresponding to these different categories. Evidence-based literature indicates that effective self-efficacy intervention is an important support for improving the oral health behaviors of patients with chronic diseases. In patients with type 2 diabetes and chronic periodontitis, integrated intervention based on the self-management model significantly enhanced the self-efficacy of the patients and continuously improved the oral health indicators (49); refined oral care intervention has also been proven to effectively improve the oral health knowledge and self-efficacy of patients with chronic periodontitis (50).

Based on the heterogeneity characteristics of these three types of patients and the evidence-based basis, we suggest that clinical treatment and care adopt a hierarchical and precise intervention strategy. For Class C1 patients, interventions should focus on supplementing basic knowledge and establishing specific habits. An empowerment education model is recommended, with one-on-one demonstration and instruction. Specifically, nurses can demonstrate correct tooth brushing techniques during patients’ dialysis sessions, provide educational booklets, and lower the cognitive threshold for skill acquisition. At the same time, cost accessibility should be considered, for example by instructing patients to choose inexpensive and effective fluoride toothpaste and appropriate mouth rinsing methods, thereby enhancing patients’ self-efficacy. Numerous studies have demonstrated that nurse-led educational interventions significantly improve patients’ adherence to disease treatment and self-management abilities, as well as substantially enhance plaque removal rates, gum health, and oral health-related self-efficacy in MHD patients (51, 52). For Class C2 patients, efforts should focus on strengthening their awareness of susceptibility to oral diseases and correcting the misconception that a healthy diet alone is sufficient for oral health. It is recommended to naturally embed oral health content into dialysis dietary education, emphasizing the importance of oral health, and helping patients transfer their existing health management motivation (centered on systemic disease) to oral health behaviors, thereby bridging the knowledge–behavior gap. It should be noted that in the actual implementation of the aforementioned intervention measures targeting vulnerable groups such as C1 and C2, the coverage of medical insurance should also be fully considered. In China, the reimbursement scope of basic medical insurance for oral treatment projects is limited. Regular oral health check-ups and basic periodontal treatment, which are preventive services, mostly require patients to pay out of pocket (53). For the Class C1 and Class C2, which are mainly composed of low-income, rural, and older adult individuals, even if they have mastered the correct oral health care skills, they may still be unable to utilize professional oral services due to the inability to bear the out-of-pocket expenses. Therefore, we suggest adding medical insurance consultation services in the nursing work and calling for including the basic oral health care of MHD patients in the medical insurance payment scope for chronic disease management, in order to truly lower the medical access threshold for vulnerable groups (54). For Class C3 patients, interventions should guide them to reconstruct their understanding of oral health, shifting from a “symptom-driven” medical-seeking pattern to a “prevention-driven” one. Nurses should play a pivotal role in the “screening - education - referral” process for patients: Make full use of the regular re-examination feature of hemodialysis patients, conduct regular oral health screenings, promptly provide oral health education to patients with oral problems, and refer patients with severe lesions to the oral department for collaborative treatment. Incorporate the oral screening program into the quarterly follow-up projects of the dialysis center, and complete it through the collaboration of professionally trained nurses or oral doctors. When oral problems are detected through screening, further explanation of the benefits of preventive dental examinations can turn into tangible benefits perceived by the patient, stimulating intrinsic motivation for regular dental visits. Such integrated treatment models contribute to the systemic health of kidney disease patients and improve their quality of life (55). It can not only enable nurses to play a core role in the continuous management of chronic diseases, but also ensure that organic oral lesions receive professional treatment.

### 4.3 Differences in oral health-related quality of life among MHD patients with different latent profiles of oral health self-efficacy

The results of this study demonstrated that there were statistically significant differences in OHRQoL scores among MHD patients with different latent profiles of oral health self-efficacy (all *p* < 0.05), indicating that OHRQoL differs across classes and that improving oral health self-efficacy levels can effectively influence patients’ OHRQoL. This finding is consistent with the study by Zhang et al. (56), which suggests that self-efficacy is an important psychological factor affecting quality of life in MHD patients. Notably, this positive association is not achieved solely through the “dental visiting behavior” pathway. Taking Class C3 as an example, although these patients had a low rate of dental visits, they maintained good daily tooth-brushing habits, exhibited high self-efficacy, and had significantly better quality of life scores than those of the other two classes. The study by Salmanpour et al. (57) also suggests that self-efficacy not only improves health outcomes by driving specific behaviors but also directly enhances quality of life by influencing individuals’ coping abilities and psychological resilience. For MHD patients, daily oral discomforts (e.g., mild pain, xerostomia, halitosis) may have a greater impact on quality of life than severe dental diseases requiring professional care. Patients with high self-efficacy are more likely to believe in their ability to cope with these discomforts, thereby effectively reducing the interference of discomfort on their mood and social interactions. In other words, self-efficacy not only improves oral health by driving specific behaviors such as tooth brushing, but also provides a psychological buffering mechanism: it gives patients a sense of control over oral problems, making them less prone to anxiety or helplessness when facing mild discomfort. In contrast, Class C1 patients have low self-efficacy and lack psychological resilience, making them more likely to catastrophize mild discomfort and adopt behavioral avoidance, which leads to prolonged problems and further reduced quality of life. This suggests to clinical practitioners that improving patients’ quality of life requires moving beyond the narrow focus of “whether they visit a dentist” and paying attention to the belief level of “whether patients believe they can manage their oral health.” Improvements in this belief alone can yield quality-of-life benefits. This phenomenon is strongly explained by social cognitive theory, which posits that human behavior is shaped by the reciprocal interaction of personal beliefs, behavior, and the environment, and is acquired and maintained through observational learning and the mediation of self-efficacy beliefs (58). The results of this study are highly consistent with this theoretical perspective, further confirming that enhancing patients’ oral health self-efficacy can directly affect their oral health behaviors and habits and indirectly improve clinical outcomes by strengthening confidence in behavioral execution.

## 5 Strengths and limitations

In this study, the latent profile analysis method was employed in the MHD patient population to identify the potential categories of oral health self-efficacy. Three clinically significant subgroups were revealed: C1 (low efficacy group), C2 (medium efficacy group), and C3 (high efficacy group). Different from variable-centered analysis methods, this person-centered approach reveals individual heterogeneity and avoids the limitation of averaging that masks group differences. This study has several limitations that may affect its internal and external validity. Firstly, a cross-sectional survey design was employed, and participants were recruited from three tertiary hospitals in Guangzhou. The geographical limitation of the sample may restrict the generalizability of the findings to patients receiving care in tertiary hospitals. Secondly, this study did not explore the longitudinal trajectory of oral health self-efficacy in MHD patients. Future research could conduct longitudinal surveys across multiple regions to validate the external generalizability of our findings and to comprehensively understand the trajectory of oral health self-efficacy in MHD patients and its impact on oral health-related quality of life (OHRQoL). What’s more, all data were collected using self-administered questionnaires based on patients’ self-perceptions, which may introduce self-report bias or recall bias. Future studies could incorporate interview methods for data collection to provide evidence for the development of personalized oral health intervention programs for MHD patients. Fourthly, the intervention strategies proposed in this study focus on behavioral changes at the patient level. However, in reality, more fundamental issues such as the improvement of the medical routine assessment system, cross-professional collaboration support, and the overall coverage of chronic disease medical insurance need to be considered.

## 6 Conclusion

This study utilized latent profile analysis to reveal the three categories and characteristics of oral health care self-efficacy in maintenance hemodialysis patients, namely C1 (low efficacy group), C2 (moderate efficacy group), and C3 (high efficacy group). It also discovered that factors such as age, place of residence, income, number of complications, brushing habits, and self-assessed oral health status significantly influenced the oral health care self-efficacy of MHD patients. Moreover, there was also heterogeneity in oral health-related quality of life among the groups. The research results indicate that enhancing patients’ oral health care self-efficacy can directly affect their healthy oral behaviors and habits, thereby improving clinical outcomes. These findings help clinicians identify high-risk groups and provide empirical evidence for implementing individualized, stepped oral health promotion programs. This study is a multi-center cross-sectional survey; future longitudinal studies are warranted to validate the stability of the class structure and to explore the long-term effects of class-based interventions on patients’ OHRQoL. In the context of a life-cycle integrated health management ecosystem, improving oral health self-efficacy in MHD patients is a complex, multifaceted task that requires collective societal efforts.

## Data Availability

The datasets used in this study are challenging to share because they contain personal health information of maintenance hemodialysis (MHD) patients, and their use is subject to strict confidentiality agreements. For privacy and ethical considerations, these data cannot be disclosed. Access to datasets is restricted, and access is only permitted if the applicable data protection regulations are met and approval is obtained from the ethics committee of the participating agency. Requests to access the datasets should be directed to the appropriate contact person (BY, 1679746965@qq.com).
